# Factors influencing utilization of intermittent preventive treatment for pregnancy in the Gushegu district, Ghana, 2013

**DOI:** 10.11604/pamj.supp.2016.25.1.6169

**Published:** 2016-10-01

**Authors:** Atasige Awin-irigu Stephen, Frederick Wurapa, Edwin Andrew Afari, Samuel Oko Sackey, Keaziah Laurencia Malm, Kofi Mensah Nyarko

**Affiliations:** 1 Ghana Field Epidemiology and Laboratory Training Program, School of Public Health, University of Ghana, Legon, Accra, Ghana; 2 Ghana Health Service, Ghana

**Keywords:** Utilization, factors, IPTp-SP, association, ANC, Gushegu

## Abstract

**Introduction:**

The coverage of adequate (≥2 doses) IPTp-SP in Ghana is below the national target of 80% and that is a threat to reducing the incidence of malaria in pregnancy. The primary objective of the study was to determine the client and facility related factors associated with adequate uptake of IPTp-SP and suggest approaches for increased uptake.

**Methods:**

A cross sectional study was conducted among ANC clients and staff in Gushegu, questionnaires was administered to 330 conveniently sampled nursing mothers and all ANC staff present. A checklist and observation were used to collect health facility data. Data was analyzed descriptively and associations between the related factors and adequate uptake of IPTp-SP were determined.

**Results:**

A total of 91.5% and 8.5% of respondents took adequate (≥2doses) and inadequate (≤1dose) IPTp-SP respectively. 85.4% respondents were early first ANC attendance and 80% were multiple gravidae. Mean ANC visits was 5.0 (standard deviation = 2.2). The key determinants for inadequate uptake of IPTp were Unemployment [OR= 4.9 95% CI (1.9-13.1], single gravidae [OR= 3.4 95% CI (1.5-7.6)] and late first ANC visit [OR= 6.8 95% CI (3.0-15.4)]. DOT practice, good staff attitude and health talk at the facility were observed and confirmed by ANC clients as satisfactory. adequate uptake of SP among respondents was high. Majorities were unemployed, have had multiple pregnancies and made early first ANC visits. Unemployment and late first ANC visits are significantly associated with taking inadequate SP dose.

**Conclusion:**

Adequate uptake of SP among respondents was high. Majorities were unemployed, have had multiple pregnancies and made early first ANC visits. Unemployment and late first ANC visits are significantly associated with taking inadequate SP dose.

## Introduction

There were an estimated 207 million cases of malaria in 2012 (uncertainty range:135 – 287 million) and an estimated 627 000 deaths globally (uncertainty range: 473 000 – 789 000) [1]. 90% of all malaria deaths occur in sub-Saharan Africa [1]. Between 2000 and 2012, the scale-up of interventions helped to reduce malaria incidence rates by 25% globally, and by 31% in the WHO African Region [1]. The effects of malaria infection during pregnancy on maternal, foetal and neonatal health are of immense health concern [2].

According to a report by the National Malaria Control Program (NMCP) Ghana, 11.3 million of Out Patient Department (OPD) malaria cases were recorded in 2013. In the same year malaria cases per 1000 population were 417 for the country and presumptively diagnosed malaria in pregnant women constituted 1.9% of all OPD cases [3].

In order to control malaria in general and in pregnant women, the WHO proposed both preventive and curative measures. They include prompt and effective management of malaria illness, the use of Insecticide Treated Nets(ITNs) and Intermittent Preventive Treatment during Pregnancy(IPTp) [4, 5]. IPTp is the administration of a single curative dose of an efficacious anti-malarial drug at least twice during pregnancy regardless of the woman’s malaria status. The drug is administered under supervision during antenatal care visits [6]. Since the implementation of IPTp, the required number of SP doses during pregnancy was three to be received between four to six months of gestation. In 2014, a new policy makes it possible for pregnant women to receive up to 5 doses of SP during pregnancy, starting as early as possible in the second trimester. IPTp-SP is recommended for all pregnant women at each scheduled antenatal care(ANC) visit until the time of delivery, provided that the doses are given at least one month apart. SP should be given during the first trimester of pregnancy: however, the last dose of IPTp-SP can be administered up to the time of delivery without safety concerns.

The clinic based approach was adopted because over 90% of pregnant women attend antenatal clinic at least once during pregnancy [6] the use ofIPTp-SP is evidently an effective method for Malaria in pregnancy control. In a study conducted in southern Ghana in 2006, after the implementation of IPTp in 2000 showed that maternal anemia had reduced by 33% and placental parasitemia reduced by 57% in 2006 as compared to 2000 [7].

In 2011, Ghana achieved a coverage of 64.4% of pregnant women receiving at least 2 doses of IPTp, this falls below the target of 80% [4] and in contrast with 84.7% Antenatal clinic (ANC) attendance coverage for at least 4 visit [4]. There is a low coverage of Intermittent Preventive Treatment using Sulphadoxinepyrimethamine (IPTp-SP) for the recommended second (IPTp2) and third (IPTp3) doses in most settings in Africa [5, 8]. Key among the factors associated with the low coverage is availability of Sulphadoxinepyrimethamine(SP) [9, 10].

From 2010-2012, out of 15127 malaria cases recorded in the Gushegu District hospital (GDH), 3234 (21.4%) were among pregnant women [11]. Despite the high levels of malaria in pregnant women, IPTp coverage was low, in 2012 only 44% of pregnant women studied at the district hospital received IPTp3 [12] and whilst in 2013 the coverage of IPTp3 among all pregnant women reported in Gushegu was 31.2% [13]. Studies identifying the factors influencing the uptake of IPTp in Gushegu to be specific and the northern region are scanty. This paper reviews the client and facility related factors that influence the uptake of the second and third doses of IPTp-Sp in the Gushegu district in the northern part of Ghana.

## Methods

### Study design

A cross-sectional study was conducted involving quantitative and qualitative data collection at the Reproductive and Child Health Unit (RCH) at Gushegu, the capital of the Gushegu district during the months of January to March 2014.

### Study population

Nursing mothers visiting the Gushegu RCH unit with infants less than 12 months old who lived in the study area for at least six months during the recent pregnancy were studied. In addition, all ANC staff present during the period of the study were interviewed for health facility data.

### Sample size

Based on a coverage of 31.2% for three doses of IPTp in the Gushegu district in 2013, 330 nursing mothers attending the RCH unit during the study period were sampled based on those available for the study (31.2% IPTP3 coverage, 95% power, p=0.05).

The coverage for three SP doses was used to determine the sample size because it was the maximum number of doses required according to the protocol for IPTp at the time of the study. Therefore adequate SP doses according to the IPTp protocol is at least 2 doses at the time of this study.

IPTp is the administration of a single curative dose of an efficacious anti-malarial drug at least twice during pregnancy regardless of the woman’s malaria status. The drug is administered under supervision during antenatal care visits [6]. Since the implementation of IPTp, the required number of SP doses during pregnancy was three to be received between four to six months of gestation.

The 330 participants available for the client level of the study were selected. All (six) ANC staff Present at the Reproductive and Child Health unit were interviewed at the facility. The participants for the client level of the study were selected randomly and based on the inclusion criteria as well as their consent to be part of the study.

### Data collection

The nursing mothers were interviewed using structured questionnaires for information on client level data. For the client level data, socio-demographic characteristics collected include age, occupation, marital status, place of residence, educational level. Place of residence refers to living within the district capital town of Gushegu or outside the capital town or other villages outside of Gushegu.

Obstetric history and ANC data collected includegravidae and timing of first ANC attendance as well as the number of ANC attendance. Structured questionnaires guided by an interviewer were administered to the nursing mothers at the RCH. The women were asked the number of ANC visits they made during pregnancy, all records were further checked from their ANC attendance book.

Antenatal clinics were observed using a checklist to document practices and verify logistics availability for the implementation of IPTp-SP. Parameters observed at the ANC included practice of Directly Observed Therapy, data recording by ANC staff, attitude of the ANC staff, availability of water for clients and the presence of educational tools and health talks on Malaria in Pregnancy (MiP). The pregnant women were asked if they got health talks and whether the ANC staff were caring and practiced DOT.

### Data analysis

Data was analyzed using Stata 11.0 statistical software. Data was summarized using frequency tables, results were reported using means, standard deviation, range and odds ratios at 95% confidence interval. The utilization of IPTp-SP among the respondents was classified adequate and inadequate, adequate was (≥2 doses) and inadequate was (≤1 dose). A bivariate logistic regression analysis was used to test the association between some of the categorical variables and the utilization of IPTp-SP.

### Ethical considerations

Ethical approval was obtained from the Ethical Review Committee of the Ghana Health Service. All participants had to consent to the study for inclusion. They were free to withdraw if they felt uncomfortable. The study population was assured that participation or otherwise will not affect the care they will receive. Respondents were identified by a unique identification and not by name.

## Results

### Demographic characteristics

Most, 197(59.9%) of the respondents were in the age group of 15-24. Majority, 264(80%) of respondents had been pregnant more than once. A very large number, 299(90.6%) lived within the township. The number of respondents without formal education compared to those with some level of it was high at 228(69.1%). Majority, 309(93.9%) of the participants lived with their partners.

### ANC visits and Utilization of IPTp-SP among respondents

Among respondents,78.7% made four or more visits to the ANC with a mean number of attendances to the ANC being 5 (range=2-7, Standard Deviation=2.2). The mean number of pregnancies among respondents was 3. The mean gestational age of the respondents at their first ANC visits was 4 months.

For the purpose of logistic analysis, women who receive one or no SP dose and two or more SP doses were categorized for analysis. As shown in Table 1, out of the 330 respondents studied, 302(91.5%) took adequate (≥2 doses) and 28(8.5%) took inadequate (≤1dose) of IPTp-SP. Receiving a second dose of SP during pregnancy is considered adequate to provide a prophylactic protection against malaria.

**Table 1:** Association between socio-demographic characteristics and IPTp Uptake

Socio-demographic Variables	(≤IPTp1) Inadequate	(≥IPTp2) Adequate	Total	OR(95%CI)
	N=28 (%)	N=302 (%)	N=330 (%)	
**Age**				
15-24 YRS	20 (74.1%)	177 (59.7%)	197(59.9%)	
25-34 YRS	6 (22.2%)	118 (38%)	124(37.7%)	0.45 (0.18-1.15)
35-44 YRS	2 (3.7%)	7 (2.4%)	9 (2.4%)	2.52 (0.49-13.01)
**Residence**				
Outside Township	2 (7.1%)	29 (9.6%)	31 (9.1%)	0.72 (0.16-3.20)
Within Township	26 (92.9%)	273 (90.4%)	299(90.6%)	
**Employment**				
Unemployed	7 (25%)	19 (6.3%)	26 (7.9%)	4.9(1.88-13.14)
Employed	21 (75%)	283 (93.7%)	304(92.1%)	
**Live with Partner**		Missing=1		
Yes	24 (86.2%)	285 (94.7%)	309(93.9%)	0.33 (0.10-1.09)
No	4 (13.8%)	16 (5.3%)	20 (6.1%)	
**Formal Education**				
None	18 (64.3%)	210 (69.5%)	228(69.1%)	0.79 (0.35-1.77)
Some	10 (35.7%)	92 (30.5%)	102(31%)	

IPTp: Intermittent Preventive Treatment for pregnancy

### Association between client factors and IPTp utilization

Association between socio-demographic characteristics

As shown in Table 1, among individuals with adequate IPTp, the majority were 15-24 years old. However this observation was not statistically significant. Among individuals with inadequate IPTp-SP only, (7.1%) lived outside the town [OR=0.7,95% CI (0.2-3.3)] and (86.2%) lived with their partners [OR=0.3 95% CI (0.1-1.1)]). A greater proportion of those with no formal education (69.5%) compared to 30.5% with some formal education took adequate IPTp-SP.

### Association between obstetric variables and IPTp utilization

In Table 2, being a multigravida was positively associated with adequate IPTp-SPamong respondents [OR=3.4 95% CI (1.5-7.6)]. Late first ANC visits were also associated with taking inadequate IPTp-SP [OR=6.8,95% CI(2.9-15.4)]. The mean gestational age of the respondents at their first ANC visits was 6 and 4 months for those who took inadequate and adequate IPTp-SP respectively.

**Table 2:** Association between obstetric and ANC history and IPTp-SP uptake

Obstetric and ANC history History	(≤IPTp1) Inadequate	(≥IPTp2) Adequate	Total	OR (95%CI)
	N=28 (%)	N=302 (%)	N=330 (%)	
**Gravidae**				
Single	12 (40.7%)	54 (18.2%)	66 (20.0%)	3.38(1.52-7.55)
Multiple	16 (59.3%)	248 (81.8%)	264(80.0%)	
**Timing of 1st ANC Visit**		Missing=3		
Late	13(48.1%)	34 (11.4%)	47 (14.6%)	6.8(2.96-15.40)
Early(≤4 gest. mths)	15(51.9%)	265(88.6%)	280(85.4%)	
**Side effects from SP in the recent pregnancy**				
Yes	10(55%)	73(24.2%)	83(25.2%)	1.74(0.77-3.94)
No	18(44.4%)	229(75.8%)	247(74.8%)	
**Malaria after SP use in the recent pregnancy**				
Yes	8(28.6%)	43(14.23%)	51(15.5%)	2.41(0.99-5.81)
No	20(71.4%)	259(85.8%)	279(84.5%)	

IPTp: Intermittent Preventive Treatment for pregnancy; ANC: Antenatal care

### ANC staff and health facility related variables

Information on ANC staff characteristics collected showed that 2 out of 6 staff had formal IPTp training, 2 out of 6 correctly defined IPTp, 5 out of 6 knew the correct start time for SP doses, all 6 knew the correct number of SP doses to receive and all 6 confirmed that ANC staff capacity was inadequate. Erratic IPTp-SP supply was admitted by all the ANC staff. It was observed at the facility that, wall posters and manuals on Malaria in Pregnancy (MiP) and IPTp were absent. Most, (87.2%) of the respondents admitted that health talks on MiP were given at the health facility. Majority (93.6%) of the respondents reported that ANC staffs were caring and polite. The practice of Directly Observed Therapy (DOT) of IPTp-SP administration was reported by 96.7% of the respondents. None of the ANC staff related variables was found to be statistically associated with IPTp-SP uptake.

Among those who took adequate IPTp, 75% did not report side effects and 85.8% did not report malaria infection during pregnancy. It was found that 75.8% and 85.8% of respondents who did not report side effects and malaria infection respectively after taking the first dose in the recent pregnancy too adequate IPTp-SP.

## Discussion

Therefore 91.5% of respondents took an adequate number of SP doses. The WHO’s recommendation emphasized that pregnant women should receive at least two doses of SP during pregnancy when the number of doses required was three.

The relationship between socio-demographic characteristics and uptake of IPTp-SP found only unemployment to be a significant predictor to taking inadequate doses ofSP. This may be attributed to the inability to afford transport fares to the ANC clinic for IPTp-SP without support from a partner or family.

A study in Tanzania, in 2008 also found that socio-demographic characteristics such asage marital status, educational level and occupation were not associated with taking a second dose of SP [14] our study showed a similar finding. This was also consistent with a previous study done in 2004, in Tanzania where individual or client factors were not found to be associated with second dose SP administration [15]. As established from this study and others, other factors rather than socio-demographic factors should be looked out for as influences to SP uptake.

However unemployment was found to be associated with inadequate uptake of SP in our study. Unemployed women are financially dependent on their spouse for transportation and other cost to attend ANC. This creates a limitation to this group of women to attend the ANC as required.

After an assessment of the obstetric and ANC history of the respondents, early first ANC visits of pregnant women was found to be significantly associated with the uptake of two or more (adequate) doses of SP in Gushegu, Ghana. This finding compares with what is known in Kenya where late first ANC attendance contributed to inadequate IPTp [10]. The mean gestational age at first ANC visit for those who took adequate IPTp-SP was 4 months, which is classified as early first ANC visit.

A total of 85.4% of the respondents in this study attended the ANC early enough (at or before 4 months gestation) this may have contributed to the high adequate uptake of SP seen in this group. This finding is similar to what was seen in a Kenyan study where 45% of the women attended their first ANC in the third trimester, only 23.7% received two doses of SP [10]. The need to promote early attendance of pregnant women to Antenatal Clinics is therefore key in ensuring adequate uptake of IPT. From the study in Gushegu (78.8%) of respondents made four or more visits to the ANC with a mean number of attendances to the ANC being 5 (Standard Deviation, SD=2.2). This figure conforms to WHO recommendations. Which states that at least three visits are made after 16 weeks of gestation, to ensure pregnant women receive at least two doses of SP [9, 8, 16].

From the study in Gushegu, Ghana women who have a first pregnancy (single gravidae) are significantly associated with taking inadequate SP dose. An experience of malaria in pregnancy among women who have been pregnant before may influence them to taking adequate SP dose as compared to primi-gravidae.

A study of the facility and staff related factors in Gushegu, Ghana showed that health talks at the ANC during clinic days which 87.2% of respondents reported attending may have imparted to them the need for ANC visits.

Findings in Tanzania indicated that attendance at health education sessions at the Maternal and Child Health (MCH) clinic was the only determining factor for IPTp-SP use among pregnant women [17]. This may have been a determinant to making required visits and receiving two or more doses of SP as found in Gushegu, Ghana. No poster on the IPTp program was seen on the walls of the health facility in Gushegu. Hence the opportunity to further educate the respondents through posters was not present although it is part of the IPTp intervention. This does not compare with what was found in a study in Tanzania, 50% of the facilities visited displayed posters explaining the purpose and benefits of IPTp [14]. This point to the poor use of posters as a means of health education.

In the facility there are other factors identified to possibly influence utilization of IPTp. The limitations in this study includes recall bias which could result from respondents not recollecting all that happened during their ANC visits in their last pregnancy. Showing samples of SP to them so they can relate their responses to the drug could have minimized the bias.

The generalizability of the study may be limited because it was carried out only in the district capital and may not be representative of the entire district hence the results have been specified to only the study site.

## Conclusion

Overall, 91.5% of respondents had received adequate IPT during pregnancy. Early first visits of pregnant women to the ANC and frequent visits significantly increase the uptake of IPTp-SP. Unemployment and being pregnant for the first time significantly reduces the uptake of IPTp-Sp. Ways to encourage early ANC attendance should be investigated by the NMCP. The study should be conducted in a more wider catchment of the Gushegu district and the northern region so that findings can be more generalized and representative.
